# Acute Pancreatitis as a Complication of a Hydatid Liver Cyst

**DOI:** 10.1155/crgm/1244948

**Published:** 2025-08-08

**Authors:** Charbel Saad, Shaza Mortada, Ali Dakroub, Makram Abou Ghaida, Lory Hage, Radwan Zahreddine, Abdallah Slim, Rodrigue Chemaly, Georges Al Hajj

**Affiliations:** ^1^Department of Surgery, Division of General Surgery, Lebanese American University Medical Center-Rizk Hospital, Beirut, Lebanon; ^2^Department of Internal Medicine, Division of Gastroenterology, Lebanese American University Medical Center-Rizk Hospital, Beirut, Lebanon; ^3^Department of Medicine, Université Paris Cité, Paris, France

**Keywords:** cholangiopancreatography, cystic echinococcosis, *Echinococcus granulosus*, endoscopic retrograde, pancreatitis

## Abstract

**Introduction and Importance:** Cystic echinococcosis is a common cystic infection in endemic areas. Solitary lesions are commonly found in the liver and are primarily caused by *Echinococcus granulosus*. Other organs can be involved as well but to a lesser degree. This paper presents a rare manifestation of a hydatid liver cyst causing acute pancreatitis.

**Case Presentation:** A 67-year-old female presented with right upper quadrant pain, nausea, and vomiting. She was found to have a large hepatic cystic echinococcosis occupying the right lobe of the liver with associated acute pancreatitis. After endoscopic retrograde cholangiopancreatography (ERCP), three small yellow membrane fragments—presumed to be daughter cyst membranes—were found obstructing the common bile duct at the ampulla of Vater. The patient underwent laparoscopic unroofing of the hepatic cystic echinococcosis and laparoscopic cholecystectomy with an uneventful postoperative course.

**Clinical Discussion:** Treatment modalities for hepatic cystic echinococcosis depend on the size of the cyst, imaging findings, cyst activity status, and associated complications. In nonendemic countries, where demographic changes occur due to travel from endemic regions, a high index of suspicion is necessary for timely diagnosis. A laparoscopic approach was chosen for its benefits related to minimally invasive surgery. While laparoscopic management of hepatic cystic echinococcosis is well described, its use in cases complicated by acute pancreatitis remains infrequently reported.

**Conclusion:** Cystic echinococcosis most commonly presents in the liver. When associated with acute pancreatitis, a combined approach involving albendazole, ERCP, and sphincterotomy is typically required, with surgical resection depending on cyst classification.

## 1. Introduction

Cystic echinococcosis is a parasitic infection caused by the tapeworm, *Echinococcus granulosus*. It holds significant public health concern in its endemic areas such as the Middle East, sub-Saharan Africa, South America, and Northern Asia particularly in populations that nurture sheep, goats, swine, and cattle [1, 2]. In these areas, the prevalence of cystic echinococcosis ranges from less than 1 to 200 cases per 100,000 [3, 4]. In nonendemic countries, cases have been reported primarily due to travel from endemic areas [2, 5].

Individuals who accidentally ingest the eggs of *Echinococcus granulosus* tapeworm are at risk for infection. In this parasite's life cycle, the definite host is usually a dog and intermediate hosts are usually sheep, goats, or camels [3, 6]. In this life cycle, humans play a role as accidental hosts, and they do not transmit the disease. Thousands of adult tapeworms can be found in the small intestine of the definite host. They are 2–7 mm long and have scolex with hooks, suckers, and three proglottid segments [7]. A brief summary of the *Echinococcus granulosus* lifecycle is available in Supporting Table 1.

Initially, cystic infection is asymptomatic. Symptoms subsequently appear due to mass effect. They can obstruct arterial, venous, or lymphatic flow and can cause complications like rupture or superimposed infections. Statistically, the liver is the most commonly affected organ, followed by the lungs [8]. Researchers have reported several irregular manifestations of cystic echinococcosis; these included the heart, breast, tibia, hand, sacrum, orbit, mediastinum, pancreas, kidney, spleen, and even brain [9–15]. This case highlights a rare complication of cystic echinococcosis, in which the hepatic cystic echinococcosis caused acute pancreatitis through biliary obstruction by migrated daughter cyst membranes. This report has been presented in accordance with the SCARE guidelines [16].

## 2. Case Presentation

A 67-year-old female known to have hypertension, treated with amlodipine 5 mg and indapamide 1.5 mg daily for the past year, presented with a 6-week history of several episodes of epigastric pain, vomiting, and nausea. She denied experiencing fever or chills and reported no specific factors associated with, exacerbating, or relieving the pain. The pain did not radiate to the back or shoulders. On physical examination, the patient had epigastric and right upper tenderness with a positive Murphy's sign and a palpable mass in the right upper quadrant region. There was good skin turgor, and no visible jaundice or scleral icterus on examination.

Vital signs on admission (Table 1) were within normal range and the patient was hemodynamically stable. She had no elevations in white blood count (WBC) but rather a neutrophil shift of 84.6% with 7.6% lymphocytes. Laboratory findings on admission (Table 1) showed elevations in gamma-glutamyl transferase (GGT), alkaline phosphatase (ALP), alanine transaminase (ALT), aspartate transaminase (AST), amylase, lipase, and direct bilirubin. Electrocardiogram (ECG) was normal and cardiac enzymes were negative.

**Figure 1:**
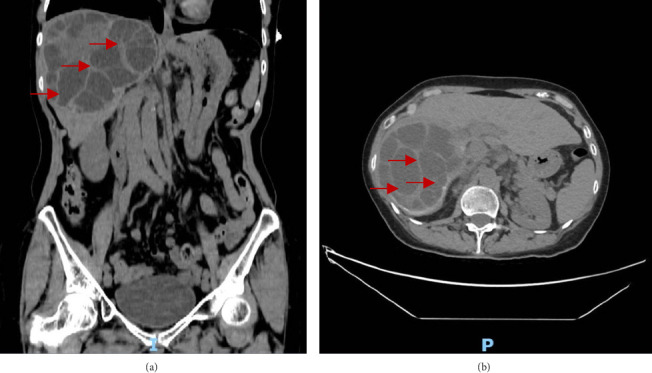
Coronal (a) and transverse (b) views of the abdomen on computerized tomography without intravenous administration of nonionic iodinated contrast media showing hepatic cyst with septations (red arrows) and mass effect.

Following an initial abdominal ultrasound that showed no cholelithiasis or biliary dilation, a contrast-enhanced computed tomography (CT) scan was obtained for further evaluation. However, the gallbladder was suboptimally distended with no evidence of sludge, stones, or pericholecystic fluid. In the liver, a large hypoattenuating cystic mass of 14 × 13 × 11 cm [transverse cut (TR) × coronal cut (CC) × anteroposterior cut (AP)] occupying the entire right lobe was seen (Figure 1). Thick septations with few peripheral rim calcifications were observed with no soft tissue component, consistent with a CE2 stage according to the WHO classification [17]. The cyst is seen almost reaching the hilum and coming in close contact with the portal trunk and lateral surface of the left portal branch with no visualization of the right posterior branch. It was compressing the adjacent hepatic artery and veins. The associated intrahepatic bile duct (IHBD) dilatation was likely attributed to the mass effect of the common hepatic duct on the bile duct confluence. The pancreas appeared mildly edematous without evidence of necrosis or peripancreatic fluid collections, consistent with a mild episode of pancreatitis. The findings were consistent with a hepatic cystic echinococcosis. No immunologic testing or PCR was performed; while diagnosis was initially based on imaging, it was confirmed intraoperatively and on histopathology. The patient was started on albendazole 400 mg twice daily, consistent with WHO recommendations. The patient lives in the Arab gulf in an urban city with minimal environmental exposure to *Echinococcus*. It was assumed that exposure occurred during her recent travel to the Middle East. The patient works in an office and has occasional alcohol intake. Her diagnosis and management occurred in an endemic area.

**Figure 2:**
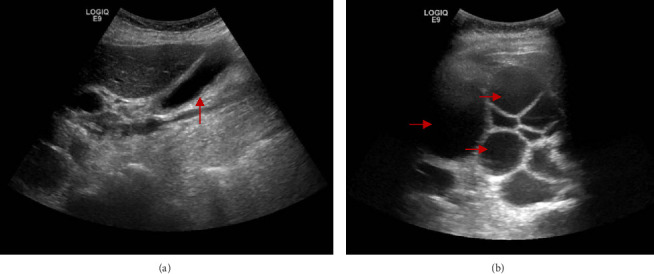
Hepatobiliary ultrasonogram showing the gallbladder (red arrow (a)) and right lobe of the liver (red arrows (b)).

Given the contradictory findings—specifically, the absence of biliary dilation on CT despite a cholestatic pattern on laboratory evaluation—a hepatobiliary ultrasonogram was performed for further assessment, as it has superior sensitivity for biliary calculi (Figure 2). It showed again no evidence of cholecystitis, cholelithiasis, or choledocholithiasis.

Owing to the cholestatic pattern on laboratory findings in addition to a surrounding context of pancreatitis, an endoscopic retrograde cholangiopancreatography (ERCP) was done to rule out migration of cyst contents such as daughter cyst membranes into the CBD. The CBD measured 7 mm with mild IHBD dilatation. After a sphincterotomy was done, three daughter cyst membranes of 3–4 mm from the hepatic cystic echinococcosis were evacuated. Complete clearance of the CBD was achieved endoscopically, and no biliary stent or nasobiliary drain was placed given the absence of persistent obstruction or biliary leak. The patient's abdominal pain improved after ERCP (Table 2); she had no further tenderness on physical examination, and she was discharged home 2 days post ERCP on albendazole 400 mg twice daily, ciprofloxacin 400 mg twice daily, and metronidazole 500 mg three times daily. The patient was to be followed up after 3 weeks of medical treatment to assess for surgical intervention.

The patient presented 3 weeks later to the emergency department with a fever of 38.7°C, concerning for acute cholangitis or an infected cyst. Laboratory evaluation revealed an elevated international normalized ratio (INR) and a reduced prothrombin time; mixing studies indicated a correction pattern consistent with a factor deficiency rather than an inhibitor. Although no definitive source was identified, clinical suspicion remained high for cyst-related infection or biliary sepsis, prompting coagulation optimization and subsequent surgical intervention. Given this picture of liver dysfunction, she received 6 units of fresh frozen plasma for coagulation optimization. Once stabilized, the patient underwent a laparoscopic unroofing of the hepatic cystic echinococcosis and a cholecystectomy. The procedure was performed electively after clinical and laboratory stabilization.

### 2.1. Laparoscopic Surgical Technique

**Figure 3:**
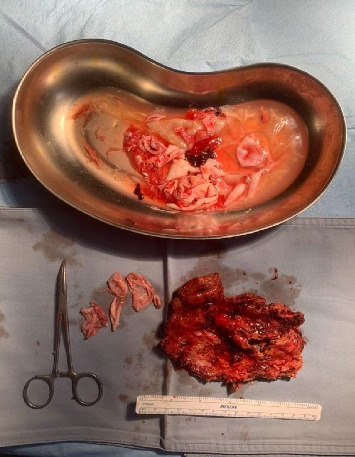
Surgical specimen.

The roof of the hepatic cystic echinococcosis was identified, and part of it was adherent to the right diaphragm. The adhesions between the liver and the diaphragm were lysed. Multiple Betadine-soaked gauze pads were introduced in a fashion to encircle the hepatic cystic echinococcosis. The latter was punctured with 200 mL drained and 200 mL of scolicidal agent (alkyltrimethylammonium bromide) injected in place. We waited for 20 min, then the cyst was opened, and clear fluid with multiple daughter cysts was suctioned. This is consistent with WHO guidelines and expert consensus recommendations for the surgical treatment of CE2 hepatic cystic echinococcosis with no biliary communication present. Using a LigaSure device, the roof of the cyst was dissected along with the adjacent liver parenchyma and was resected. The specimen along with many large daughter cysts was retrieved using an endobag from the left abdominal mid-clavicular incision. No biliary leak was identified from the remaining cyst wall. The surgical specimen can be seen in Figure 3.

**Figure 4:**
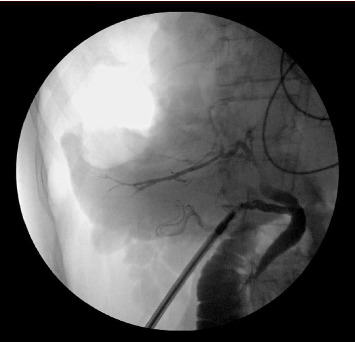
Intraoperative cholangiogram showing a patent biliary tree with no signs of contrast extravasation.

Due to an anatomical contiguity and dense adhesions observed between the cyst and the gallbladder, it was decided to conduct a cholecystectomy. The gallbladder was retracted cephalically. Using monopolar electrocautery, the visceral peritoneum was dissected just above the level of the cystic pedicle. Continued dissection was carried out for identification of the cystic artery and duct. Critical view of safety was achieved. The cystic artery was clipped and transected. The cystic duct was milked and then clipped proximal to the gallbladder neck. Intraoperative cholangiogram was performed prior to scolicidal agent injection to confirm the absence of cystobiliary communication (Figure 4). Contrast reached the duodenum and there were no signs of contrast extravasation into the hepatic cystic echinococcosis. No hydatid material was observed within the biliary tree during cholangiogram. The cystic duct was then clipped distally and transected. The gallbladder was densely adherent to the liver bed in keeping with an intrahepatic gallbladder. It was freed from the liver bed using electrocautery and removed from the abdomen. Importantly, although the cyst was in close anatomical proximity to the gallbladder, no cystobiliary communication was observed on intraoperative cholangiogram, supporting the safe intraoperative use of a scolicidal agent.

No postoperative complications were noted, and the diagnosis was confirmed on the pathology report. On day 4 postoperatively, albendazole dosage was adjusted to 200 mg twice daily and she was discharged home on the same dose for 3 months. This approach aligns with existing literature suggesting preoperative and postoperative albendazole therapy to decrease risk of recurrence and inactivate viable cysts [17]. She was followed up after 1 month, 3 months, and 6 months postoperatively and has had no recurrence or relapse to date. Although the patient remained asymptomatic with normalized labs, follow-up imaging such as magnetic resonance cholangiopancreatography (MRCP) was not performed, which is a limitation given that recurrences may be subclinical.

## 3. Discussion

Cystic echinococcosis usually presents in the liver as an asymptomatic mass. However, hepatic echinococcosis can present with complications such as acute pancreatitis by communicating with the biliary tree. This has been suggested to be best managed by ERCP and sphincterotomy [18–20], as has been the case with our patient. However, the risk of rupture of the cyst or recurrence of symptoms will likely persist if not for medical or surgical treatment or both [21].

The relationship between hydatid cysts and acute pancreatitis is typically mediated by cystobiliary communication. When daughter cyst membranes or other hydatid materials migrate into the biliary tree, they may obstruct the common bile duct or the ampulla of Vater, resulting in upstream pancreatic duct obstruction and enzymatic activation. This triggers a cascade of inflammatory responses consistent with acute pancreatitis. Less commonly, external compression of the biliary tree by a large cyst may also contribute to biliary stasis and secondary pancreatic inflammation. In either case, mechanical obstruction is the unifying mechanism linking hepatic hydatid cysts to pancreatic injury [21–23].

In our patient, daughter cyst membranes were retrieved from the common bile duct during ERCP, implicating intrabiliary migration as the most likely cause of acute pancreatitis. While direct compression of the biliary tract by the cyst remains a potential mechanism, the resolution of symptoms following endoscopic intervention supports obstructive migration. The staged approach, initial decompression followed by delayed surgery, allowed for clinical stabilization and reduced operative risk.

Surgical management of hepatic echinococcosis varies by cyst characteristics. Radical procedures include hepatic resection and pericystectomy, while conservative techniques include unroofing, drainage, and omentoplasty. For CE2-stage cysts with multiple daughter cysts, unroofing is often preferred. This approach is supported by WHO guidelines and expert consensus for CE2 cyst management [17, 24]. In our case, a laparoscopic approach was safely performed after stabilization. Although concerns such as anaphylaxis and biliary leaks exist with laparoscopy [25, 26], evidence suggests comparable outcomes to open surgery, with benefits like reduced hospital stay and faster recovery [22, 27–30].

While percutaneous techniques (PAIR) are an option for certain active cysts, surgery remains the gold standard when there is risk of rupture, communication with bile ducts, or large cyst burden [17]. In our case, the cyst's location and size, along with biliary involvement, made surgical resection necessary. Interestingly, although hydatid debris was found in the CBD during ERCP, no cystobiliary fistula was identified intraoperatively. This suggests the presence of a transient microfistula that may have sealed spontaneously, or temporary migration of cyst contents under elevated biliary pressure. Such occurrences have been described in prior reports and highlight the dynamic nature of cystobiliary communication in echinococcosis [22, 23, 31]. Migration of cyst contents into the bile duct may obstruct the ampulla of Vater or pancreatic duct, triggering inflammation. However, no clear guidelines exist for management. Our case contributes to the growing evidence supporting a two-stage approach: ERCP to manage acute pancreatitis, followed by laparoscopic cystectomy for definitive treatment.

### 3.1. Limitations

This case report is limited by the absence of inflammatory markers, such as C-reactive protein, at initial presentation, which may have aided in assessing the severity of pancreatitis. In addition, images from the ERCP were unavailable for review or publication. Although the patient remained asymptomatic with normalized laboratory values, follow-up imaging such as MRCP was not obtained, limiting the ability to detect potential subclinical recurrence.

## 4. Conclusion

Cystic echinococcosis is an important infectious concern not just in endemic countries but also in countries with significant travel from endemic areas. Therefore, diagnosis and early treatment are essential in preventing related complications. The liver is the most affected organ, and in some cases, the cyst can cause acute pancreatitis. This may result from daughter cyst migration or obstructive compression due to the cyst. The treatment approach includes albendazole, ERCP, and sphincterotomy. Surgical intervention may be beneficial depending on the classification of the cyst. However, the optimal surgical approach is yet to be studied and determined.

## Figures and Tables

**Table 1:** Vital signs and laboratory findings on admission.

Parameter	Value	Normal range
Blood pressure	122/74 mmHg	—
Heart rate	65 bpm	—
Temperature	37.2°C	—
Oxygen saturation (SpO_2_)	100%	—
Glycemia	113 mg/dL (6.3 mmol/L)	70–100 mg/dL (3.9–5.6 mmol/L)
Gamma-glutamyl transferase (GGT)	814 U/L	< 40 U/L
Alkaline phosphatase (ALP)	394 U/L	35–105 U/L
Alanine transaminase (ALT)	114 U/L	< 34 U/L
Aspartate transaminase (AST)	86 U/L	< 33 U/L
Amylase	1170 U/L	28–100 U/L
Lipase	1348 U/L	13–60 U/L
Direct bilirubin	3.1 mg/dL (53 μmol/L)	0–0.3 mg/dL (0–5.1 μmol/L)
Indirect bilirubin	0.6 mg/dL (10 μmol/L)	0.2–1.0 mg/dL (3.4–17 μmol/L)
Total bilirubin	3.7 mg/dL (63 μmol/L)	0.2–1.0 mg/dL (3.4–17 μmol/L)

**Table 2:** Laboratory findings before and after ERCP.

Parameter	Normal range	Pre-ERCP	Post-ERCP
Gamma-glutamyl transferase (GGT)	< 40 U/L	879 U/L	655 U/L
Alkaline phosphatase (ALP)	35–105 U/L	444 U/L	343 U/L
Alanine transaminase (ALT)	< 34 U/L	80 U/L	52 U/L
Aspartate transaminase (AST)	< 33 U/L	61 U/L	52 U/L
Lipase	13–60 U/L	152 U/L	34 U/L
Direct bilirubin	0–0.3 mg/dL (0–5.1 μmol/L)	4.7 mg/dL (80 μmol/L)	1.8 mg/dL (31 μmol/L)
Indirect bilirubin	0.2–1.0 mg/dL (3.4–17 μmol/L)	0.3 mg/dL (5 μmol/L)	0.4 mg/dL (7 μmol/L)
Total bilirubin	0.2–1.0 mg/dL (3.4–17 μmol/L)	5.0 mg/dL (85 μmol/L)	2.2 mg/dL (38 μmol/L)

## Data Availability

Data sharing is not applicable to this article as no new data were created or analyzed in this study.

## Supporting Information

Supporting Table 1: summary of the *Echinococcus granulosus* lifecycle, including definitive and intermediate hosts, transmission dynamics, and developmental stages of the parasite.
