# Supplementary material for: Acute Pancreatitis as a Complication of a Hydatid Liver Cyst
Source: Case Rep Gastrointest Med. 2025 Aug 8;2025:1244948. doi: 10.1155/crgm/1244948 (PMC12356676; doi:10.1155/crgm/1244948)
Supplement: Supporting Information — Additional supporting information can be found online in the Supporting Information section. [file 1244948.f1.docx]

| **Egg Stage** | The adult tapeworm releases the eggs into the small intestine of the definitive host. |
| --- | --- |
| **Larval Stage** | The eggs leave the host’s body via the feces. These contaminate the surrounding environment, and when a herbivorous intermediate host ingests these contaminated eggs, they hatch in the intestine and release oncospheres. These are larvae with infective potential. They penetrate the intestinal wall and spread hematogenously or lymphatically. |
| **Hepatic cystic echinococcosis Stage** | After they reach a trophic organ, they develop into growing cysts. Inside the cyst are maturing larvae. This process can take several months or years. |
| **Definitive Host Stage** | The intermediate host is frequently a prey of the carnivorous definitive host. Upon exposure of the hydatid cysts to the digestive juices of the stomach, the larvae envaginate their scolex and adhere to the wall of the intestine where it can grow to several meters long.  This is where the cycle repeats itself. |

**Supplementary Table 1.** Summary of the Echinococcus granulosus lifecycle, including host roles, transmission, and cyst development stages.
